# Molecular epidemiology of extended-spectrum β-lactamase-producing *Escherichia coli* in the community and hospital in Korea: emergence of ST131 producing CTX-M-15

**DOI:** 10.1186/1471-2334-12-149

**Published:** 2012-06-29

**Authors:** Sun Hee Park, Ji-Hyun Byun, Su-Mi Choi, Dong-Gun Lee, Si-Hyun Kim, Jae-Cheol Kwon, Chulmin Park, Jung-Hyun Choi, Jin-Hong Yoo

**Affiliations:** 1 Division of Infectious Diseases, Department of Internal Medicine, The Catholic University of Korea, College of Medicine, Seoul, Korea; 2 Catholic Research Institutes of Medical Science, The Catholic University of Korea, College of Medicine, Seoul, Korea; 3 Division of Infectious Diseases, Department of Internal Medicine, Korea University Medical College, Seoul, Korea; 4 Division of Infectious Diseases, Department of Internal Medicine, Ilsan Hospital, Goyang-si, Gyeonggi-do, Korea; 5 Department of internal medicine, Bucheon St Mary’s Hospital, The Catholic University of Korea, #2, Sosa-Dong, Wonmi-Gu, Bucheon, Gyeonggi-Do 420-717, Republic of Korea

**Keywords:** *Escherichia coli*, Extended-spectrum β-lactamse, Molecular epidemiology, Community, Korea

## Abstract

**Background:**

The prevalence of extended-spectrum β-lactamase (ESBL)-producing *Escherichia coli* has been increased not only in the hospital but also in the community worldwide. This study was aimed to characterize ESBL- producing *E. coli* isolates and to investigate the molecular epidemiology of community isolates in comparison with hospital isolates at a single center in Korea.

**Methods:**

A total of 142 ESBL-producing *E. coli* isolates were collected at Daejeon St Mary’s Hospital in Korea from January 2008 to September 2009. The ESBLs were characterized by PCR sequencing using specific primers. The genetic relatedness was determined by pulsed field gel electrophoresis (PFGE) and multilocus sequence typing (MLST).

**Results:**

Of 142 isolates, 139 were positive for CTX-M type ESBLs; CTX-M-14 (n = 69, 49.6 %), CTX-M-15 (n = 53, 38.1 %) and both CTX-M-14 and -15 (n = 17, 12.2 %). CTX-M-14 and CTX-M-15 were detected in both community and hospital isolates whereas isolates producing both CTX-M14 and-15 were mainly identified in the hospital. CTX-M producing *E. coli* isolates were genetically heterogeneous, revealing 75 distinct PFGE types. By MLST, 21 distinctive STs including 5 major STs (ST131, ST405, ST38, ST10, and ST648) were identified. Major STs were distributed in both community and hospital isolates, and ST131 was the predominant clone regardless of the locations of acquisition. No specific major STs were confined to a single type of ESBLs. However, ST131 clones were significantly associated with CTX-M-15 and the majority of them were multidrug-resistant. Distinctively, we identified a hospital epidemic caused by the dissemination of an epidemic strain, ST131-PFGE type 10, characterized by multidrug resistance and co-producing both CTX-Ms with OXA-1 or TEM-1b.

**Conclusions:**

The epidemiology of ESBL-producing *E. coli* is a complex and evolving phenomenon attributed to the horizontal transfer of genetic elements and clonal spread of major clones, predominantly ST131. The multidrug resistant ST131 clone producing CTX-M-15 has emerged as a major clone in both the community and hospital, suggesting the widespread of this epidemic clone in Korea.

## Background

In recent years, there has been a dramatic increase in the prevalence of *Escherichia coli* that produce extended-spectrum β-lactamases (ESBLs) in the community and in the hospital [1]. Among the various ESBLs produced by *E. coli*, the CTX-M family currently predominates and substitutes the classic SHV and TEM types. Within this family, CTX-M-15 has become the most widely distributed in the world. Furthermore, *E. coli* strains producing CTX-M-15, which are generally multidrug resistant, have emerged as an important pathogen causing both community- and hospital-onset infections. The successful dissemination of CTX-M-15 was considered to be due to dissemination of genetic elements and clonal expansion of a pandemic *E. coli* clone ST131 with a high virulent potential [2-4]. The *E. coli* ST131 clone producing CTX-M-15 was characterized by multidrug resistance and co-production of OXA-1 or TEM-1b β-lactamases as well as *aac(6’)-Ib-cr*. In early reports, ST131 clone producing CTX-M-15 was regarded to cause predominantly community-onset infections [5-7]. However, this clone has been increasingly identified in the healthcare settings [8,9].

The distribution of predominant CTX-M type ESBLs varies geographically [10]. In Korea, CTX-M-14 had been reported to be the dominant CTX-M type in *E. coli* since the first report on CTX-M-14 in 2001[11,12]. However, CTX-M-15-producing *E. coli* isolates have been increasingly detected in Korea as in many other countries [13-15]. Furthermore, ST131 clone has emerged as a major *E. coli* clone among ciprofloxacin-resistant *E. coli* isolates, which causes community-onset urinary tract infections in Korea [16]. The epidemiology of *E. coli* has been continuously evolving on a global scale and there has been a dramatic increase in ESBL-producing *E. coli* in the community, let alone in the healthcare settings. Since 2007, we have observed the rapid increase in ESBL-producing *E. coli* as a cause of community-onset bacteremia at this center as well as other Korean hospitals [15,17,18]. It needs to be elucidated the molecular characteristics of ESBL-producing *E. coli* community isolates in comparison with the hospital isolates in Korea.

The objectives of this study were to characterize ESBLs produced by *E. coli* isolates causing community and hospital onset infections, and to compare the molecular characteristics and epidemiology between the hospital and the community isolates at this center in Korea.

## Methods

### Study design and Patients

This was a retrospective cohort study of all consecutive patients who were infected or colonized by ESBL-producing *E. coli* isolates, and who attended Daejeon St Mary’s Hospital between January 2008 and September 2009. Daejeon St Mary’s Hospital is a 560-bed university-affiliated, secondary care, community hospital located in Daejeon, South Korea, which has a population of 1.5 million people. The hospital receives around 20,450 admissions annually. The clinical microbiology laboratory at Daejeon St Mary’s Hospital only receives clinical samples from hospitalized patients, and from patients attending the emergency department or outpatient clinics of this hospital. All of the patients in whom ESBL-producing *E. coli* isolates were recovered were identified by retrieving the microbiology data from the clinical microbiology laboratory. Clinical data, including demographic features, co-morbidities, types of infection, locations of acquisition, and healthcare-associated risk factors, were obtained by reviewing the patients' medical records. A total of 142 consecutive ESBL-producing *E. coli* isolates were collected from 202 episodes of ESBL producing *E. coli* infection or colonization during the study period. Only the first isolate from each patient was included in this study. The study was approved by the ethics committee of Daejeon St Mary's hospital, the Catholic University of Korea (IRB code: DC 10ENSI001). The ethics committee waived the requirement for informed consent because this study involved minimal risk to the patients, and the waiver would not adversely affect the rights or welfare of the patients.

### Antimicrobial susceptibility tests and confirmation of ESBL production

Species identification and susceptibility tests were performed with the MicroScan NegCombo Panel Type 32 (Dade Behring, Sacramento, CA, USA) in accordance with the manufacturer’s instruction. In addition, in vitro antimicrobial susceptibility testing was performed by the broth microdilution method and the results were interpreted by using the 2010 Clinical Laboratory Standard Institute (CLSI) breakpoints [19]. ESBL production was detected by using the 2009 CLSI recommendations for ESBL screening and confirmation tests [20]. Double disk synergy test was performed for a confirmation test. In brief, disks containing 30 μg of cefotaxime and ceftazidime, either alone or coupled with 10 μg of clavulanate, (Oxoid Ltd, Cambridge, UK), were placed at distances of 20 mm (center to center). When the inhibition zone differed by ≥5 mm, between at least 1 of the combination disks and its corresponding single antibiotic disk, the strain was identified as an ESBL producer. *E. coli* ATCC 25922 and *Klebsiella pneumoniae* ATCC 700603 were used as reference strains. Isolates were considered multidrug resistant (MDR) if they were non-susceptible to at least one agent in ≥3 antimicrobial classes[21]. The number of antimicrobial classes to which the isolates were non-susceptible, apart from penicillin and cephalosporin, was counted and denoted as the resistance score for further characterization of the antibiotics resistance profiles of each isolate [22].

### Molecular characterization of β-lactamases

Polymerase chain reactions (PCRs) and sequence analyses were conducted to determine the gene responsible for the ESBL phenotype in the ESBL producers. PCRs for *bla*_*TEM*_*, bla*_*SHV*_*, bla*_*OXA-1*_*,* and *bla*_*CTX-M*_ genes were conducted by using PCR primers and the conditions as previously described [12,23,24]. Automatic sequencing was performed on both strands of all PCR products with the ABI Prism 3700 DNA SEQUENCER (Applied Biosystem, Foster City, CA). The types of β-lactamase genes were identified by comparing the sequences of the database of G. Jacoby and K. Bush (http://www.lahey.org/Studies/) and the sequences in GenBank (http://blast.ncbi.nlm.nih.gov/Blast.cgi).

### Pulsed-field gel electrophoresis

ESBL-producing *E. coli* isolates were typed with pulsed-field gel electrophoresis (PFGE) after the extraction of genomic DNA and digestion with *Xba*I by using the standardized protocol [25]. PFGE was performed using a CHEF-DR III apparatus (Bio-Rad Korea, Seoul, Korea). The digital images were analyzed with Fingerprinting II Informatix software (Bio-Rad, Hercules, CA, USA), using the Dice coefficient, and UPGMA with a 1 % tolerance and a 0.5 % optimizing setting value. Isolates were considered to be genetically related if the Dice coefficient correlation was ≥80 %, which corresponds to the ‘possibly related criteria’ of Tenover at al. [26].

### Multilocus sequence typing

Multilocus sequence typing (MLST) was conducted as previously described, based on seven housekeeping genes (*atpA, fumC, gyrB, icd, mdh , purA, and recA*). Different sequences of a given locus were assigned an allele number based on *E. coli* MLST database (http://mlst.ucc.ie/dbs/Ecoli) and each unique combination of alleles (the allelic profile) was designated as a sequence type (ST) [27].

### Definitions

The type of infection was defined according to the Centers for Disease Control and Prevention (CDC)/National Healthcare Safety Network (NHSN) criteria [28]. Patients who failed to meet the criteria for infection were considered to be colonized.

*E. coli* infections were defined as a community-onset (CO) when a positive culture was obtained at the time of hospital admission or < 48 h after hospitalization. Hospital-onset (HO) infections were defined as an infection diagnosed after hospitalization for 48 h or more. In addition, the patients who had been hospitalized within 2 weeks prior to admission or who had been transferred from other hospitals were also defined as having hospital-onset infections. Episodes of community-onset infections were classified as healthcare-associated (HA), if any of the following criteria were present: 1) the receipt of intravenous therapy, nursing care at home or in a day hospital during the 30 days before infections, including the performance of urinary or digestive tract endoscopy or other invasive procedures; 2) attendance to a hospital or hemodynamic clinic within the 30 days before infections; 3) hospitalization for >2 days in an acute care hospital or residence in a nursing home or long term care facility during the year before infections. Otherwise, the cases were considered to be strictly community-associated (CA) [29,30]. *E. coli* colonizations were also epidemiologically categorized as defined above.

### Statistical analysis

Data analysis was performed using SPSS software, version 12.0 (SPSS). Statistical significance was assessed via the χ^2^ test or Fisher’s exact test for the categorical variables and Student’s t-test or the Mann–Whitney U-test for the continuous variables. Odds ratios (ORs) and 95 % confidence intervals (CIs) were calculated. All *P*-values were two-tailed and a *P*-value of <0.05 was considered statistically significant.

## Results

During the study period, a total of 202 episodes of ESBL producing *E. coli* infection or colonization were identified, accounting for 17.3 % (202/1170) of all *E. coli* infections/colonizations. Among 202 episodes, 95 were classified as hospital-onset and 107 as community-onset, including 45 cases of community-associated and 62 of healthcare-associated. The percentage of community-associated infection/colonization caused by ESBL producers was 3.8 % of all *E. coli* infections/colonizations. The demographics and clinical characteristics of 202 patients are summarized in Table 1.

**Table 1:** **Demographics and clinical characteristics of patients infected or colonized by extended-spectrum β-lactamase producing* Escherichia coli ***

	**Total episodes (n = 202)**	**Hospital-onset (n = 95)**	**Community-onset**		
			**Total (n = 107)**	**Healthcare-associated (n = 62)**	**Community-associated (n = 45)**
Age, year, mean (range)	60 (0–97)	68.3 (5–97)	52.7 (0–85)	64.3 (0–85)	36.7 (0–85)
Gender, M: F	79:123	50:45	29:78	19:43	10:35
Healthcare-associated risk factors					
Previous Admission	89 (44.1)	41 (43.2)	48 (44.9)	48 (77.4)	NA^a^
History of long term care facility stay	34 (16.8)	20 (21.1)	14 (13.1)	14 (22.6)	NA^a^
Hemodialysis	16 (7.9)	9 (9.5)	7 (6.5)	7 (11.3)	NA^a^
Surgery/invasive procedures	56 (27.7)	46 (48.4)	10 (9.3)	10 (16.1)	NA^a^
Co-morbidity					
Cerebrovascular disease	85 (42.1)	63 (66.3)	22 (20.6)	18 (29.0)	4 (8.9)
Diabetes	68 (33.7)	40 (42.1)	28 (26.2)	22 (35.5)	6 (13.3)
Chronic kidney disease	48 (23.8)	17 (17.9)	31 (29.0)	25 (40.3)	6 (13.3)
Malignancy	40 (19.8)	22 (23.3)	18 (16.8)	16 (25.8)	2 (4.4)
Heart failure	27 (13.4)	20 (21.1)	7 (6.5)	5 (8.1)	2 (4.4)
Chronic lung disease	18 (8.9)	15 (15.8)	3 (2.8)	2 (3.2)	1 (2.2)
Liver cirrhosis	14 (6.98)	7 (7.4)	7 (6.5)	7 (11.3)	0 (0)
Transplant	6 (3.0)	3 (3.2)	3 (2.8)	1 (1.6)	2 (4.4)
Obstructive uropathy	18 (8.9)	1 (1.1)	17 (15.9)	12 (19.4)	5 (11.1)
Vascular catheter	58 (28.7)	55 (57.9)	3 (2.8)	3 (4.8)	0 (0)
Urinary catheter	94 (46.5)	76 (80)	18 (16.8)	18 (29)	0 (0)
Recent use of antibiotics	141 (69.8)	88 (92.6)	53 (49.5)	34 (54.8)	19 (69.8)
Infection: colonization	105:97	34:61	71:36	41:21	30:15
Type of infection					
Bloodstream infection	23 (11.4)	4 (11.8)	19 (26.8)	12 (29.3)	7 (23.3)
Urinary tract infection	49 (24.3)	5 (14.7)	44 (44)	23 (56.1)	21 (70)
Lung infection	20 (9.9)	17 (50)	3 (4.2)	3 (7.3)	0 (0)
Intra-abdominal infection	5 (4.8)	5 (14.7)	1 (1.4)	1 (2.4)	0 (0)
SST^b^/bone infection	5 (6.7)	3 (8.8)	4 (5.6)	2 (4.9)	2 (6.7)
Colonization site					
Urine	56 (54.6)	28 (45.9)	25 (69.4)	13 (61.9)	12 (80)
Respiratory tract	23 (23.7)	20 (32.8)	3 (8.3)	3 (14.3)	0 (0)
Wound	8 (8.2)	8 (13.1)	0 (0)	0 (0)	0 (0)
Bile	4 (4.1)	1 (1.6)	3 (8.3)	3 (14.3)	0 (0)
Others	9 (9.3)	4 (6.6)	5 (13.9)	2 (9.5)	3 (20)
No. of isolates collected	142 (70.3)	61 (64.2)	81 (75.7)	46 (74.2)	35 (77.8)

Data are no. (%) of patients, unless otherwise indicated.

^a^NA, not applicable.

^b^SST, skin and soft tissue infection.

A total of 142 isolates were collected for further study, consisting of 61 hospital isolates and 81 community isolates (35 CA and 46 HA isolates). Isolates were recovered from urine (n = 72, 50.7 %), respiratory specimen (n = 35, 26.6 %), blood (n = 12, 8.5 %), wound/pus (n = 14, 9.9 %), and intra-abdominal specimen such as bile or peritoneal fluid (n = 9, 6.3 %).

### β-lactamases of ESBL-producing *E. coli*

Of the 142 *E. coli* isolates with ESBL phenotype, 139 were tested positive for ESBL production and no ESBL genes were detected in 3 isolates. All ESBL types belonged to the CTX-M family, whereas TEM or SHV type ESBL genes were not detected. Among 139 isolates producing CTX-M type ESBLs, 69 isolates (49.6 %) produced CTX-M-14, 53 (38.1 %) produced CTX-M-15, and 17 (12.2 %) produced both CTX-M-14 and CTX-M-15 (Table 2). Other β-lactamases including OXA-1 and TEM-1b were concomitantly produced by 35.9 % and 72.5 % of all isolates, respectively. SHV-1 was detected in only one isolate. The co-production of OXA-1 was significantly associated with CTX-M-15, either alone (29/53, 54.7 %; *P* < 0.001) or coupled with CTX-M-14 (13/17, 76.5 %; *P* < 0.001), compared to CTX-M-14 alone (9/69, 13.0 %). CTX-M-14 and CTX-M-15 both were identified in community isolates and hospital isolates, and the proportion of CTX-M-14 and CTX-M-15-producing isolates was not significantly different in both the community and the hospital. However, *E. coli* isolates producing both CTX-M-14 and CTX-M-15 were mainly hospital-onset (16/17, 94.1 %) and the remaining one isolate was recovered from a patient with healthcare-associated risk factors (a nursing home resident). Hospital isolates were more likely to produce OXA-1 (29/60, 48.3 %) than community ones (22/79, 27.8 %) (*P* = 0.013, OR 2.42, 95 % CI 1.20-4.91).

**Table 2:** **Types of β-lactamases produced by extended-spectrum β-lactamase (ESBL)-producing* Escherichia coli *according to the locations of acquisition.**

**β-lactamase**	**Total (n = 139)**	**Hospital-onset (n = 60)**	**Community-onset**		
			**Total (n = 79)**	**Healthcare-associated (n = 46)**	**Community-associated (n = 33)**
CTX-M-14	69 (49.6)	24 (40)	45 (56.9)	23 (60.0)	22 (66.7)
CTX-M-14	17	7	10	6	4
CTX-M-14 + TEM-1b	43	14	29	15	14
CTX-M-14 + OXA-1	1	0	1	0	1
CTX-M-14 + TEM-1b + OXA-1	8	3	5	2	3
CTX-M-15	53 (38.1)	20 (33.3)	33 (41.8)	22 (47.8)	11 (33.3)
CTX-M-15	8	2	6	3	3
CTX-M-15 + TEM-1b	16	4	12	7	5
CTX-M-15 + OXA-1	10	3	7	4	3
CTX-M-15 + TEM-1b + OXA-1	19	11	8	8	0
CTX-M-14 + M-15	17 (12.2)	16 (26.7)	1 (1.3)	1 (2.2)	0 (0)
CTX-M-14 + M-15 + TEM-1b	4	4	0	0	0
CTX-M-14 + M-15 + OXA-1	1	1	0	0	0
CTX-M-14 + M-15 + TEM-1b + OXA-1	11	10	1	1	0
CTX-M-14 + M-15 + TEM-1b + OXA-1 + SHV-1	1	1	0	0	0

Data are no. (%) of isolates, unless otherwise indicated.

### Antimicrobial susceptibilities

Antimicrobial resistance profiles of ESBL-producing *E. coli* are summarized in Table 3. Of all 139 isolates identified to produce CTX-M type ESBLs, 105 isolates (75.5 %) were non-susceptible to ciprofloxacin, 14 (10.1 %) to amikacin, 92 (65.5 %) to gentamicin, and 94 (67.6 %) to tobramycin, 33 (23.7 %) to piperacillin-tazobactam, 89 (64.0 %) to trimethoprim-sulfamethoxazole. None of the isolates were non-susceptible to imipenem or meropenem. Overall, 120 isolates (86.3 %) were defined as multidrug resistant. Hospital isolates showed a higher non-susceptibility rate to ciprofloxacin, gentamicin, and piperacillin-tazobactam than did community isolates.

**Table 3:** **Antimicrobial resistance profiles of CTX-M-producing* Escherichia coli *according to locations of acquisition and CTX-M types**

**Antimicrobial agent**	**Total (n = 139)**	**Hospital-onset (n = 60)**	**Community-onset**		**CTX-M type**			
			**Total (n = 79)**	**Healthcare-associated (n = 46)**	**Community-associated (n = 33)**	**CTX-M-14 (n = 69)**	**CTX-M-15 (n = 53)**	**CTX M-14 + M-15 (n = 17)**
Amikacin	14 (10.1)	7 (11.7)	7 (8.9)	3 (6.5)	4 (12.1)	4 (5.8)	7 (13.2)	3 (17.6)
Gentamicin	92 (65.5)	45 (73.8) ^a^	47 (59.5)	28 (60.9)	19 (57.6)	37 (53.6)	40 (75.5)^b^	15 (88.2)^b^
Tobramycin	94 (67.6)	46 (76.7)	48 (60.8)	29 (63.0)	19 (57.6)	36 (52.2)	43 (81.1)^b^	15 (88.2) ^b^
Aztreonam	130 (93.5)	60 (100)	70 (88.6)	43 (93.5)	27 (81.8)	62 (89.9)	51 (96.2)	17 (100)
Cefepime	132 (95.0)	58 (96.7)	74 (93.7)	44 (95.7)	30 (90.9)	65 (94.2)	51 (96.2)	16 (94.1)
Ceftazidime	77 (55.4)	38 (63.9)	39 (49.4)	24 (52.2)	15 (45.5)	20 (29.0)	43 (81.1)^b^	14 (82.4) ^b^
Cefotaxime	139 (100)	60 (100)	78 (100)	46 (100)	32 (100)	69 (100)	52 (100)	17 (100)
Ciprofloxacin	105 (75.5)	52 (86.7) ^a^	53 (67.1)	34 (73.9)	19 (57.6)	47 (68.1)	42 (79.2)	16 (94.1)^b^
Piperacillin-tazobactam	33 (23.7)	20 (33.3) ^a^	13 (16.5)	7 (15.2)	6 (18.2)	11 (15.9)	10 (18.9)	12 (70.6) ^b,c^
Trimethoprim-sulfamethoxazole	89 (64.0)	38 (63.3)	51 (64.6)	29 (63.0)	22 (66.7)	52 (75.3)	31 (58.5) ^b^	6 (35.3) ^b^
Multidrug resistance	120 (86.3)	53 (88.4)	67 (84.8)	39 (84.8)	28 (84.8)	55 (79.7)	48 (90.6)	17 (100)^b^
Resistance score^d^ ≥ 3	71 (51.1)	39 (65.0) ^a^	32 (40.5)	19 (41.3)	13 (39.4)	26 (37.7)	31 (58.5) ^b^	14 (82.4) ^b^

^a^ For comparison with CO isolates, *P* < 0.05.

^b^ For comparison with CTX-M-14 producing isolates, *P* < 0.05.

^c^ For comparison with CTX-M-15 producing isolates, *P* < 0.001.

^d^ Number of antimicrobial classes, apart from penicillin and cephalosporin, to which *E. coli* isolates are non-susceptible.

Antibiotics resistance patterns were different according to the types of CTX-M produced by the isolates (Table 3). CTX-M-15 producing *E. coli* had propensity to be more non-susceptible to multiple antibiotics than CTX-M-14 producers. Isolates producing both CTX-M-14 and -15 showed the highest MDR rate (17/17, 100 %,), being more likely to be non-susceptible to gentamicin, tobramycin, ciprofloxacin, and pipercillin-tazobactam, and ceftazidime. Likewise, *E. coli* isolates producing CTX-M-15 alone were more likely to be non-susceptible to gentamicin, tobramcyin, and ceftazidime than CTX-M-14 producers. In contrast, the non-susceptibility rate to trimethoprim-sulfamethoxazole was lower in CTX-M-15 producing isolates than in isolates producing CTX-M-14 alone.

### Multilocus sequence typing

Among the total of 130 isolates studied, MLST analysis identified 21 distinctive STs (Table 4). The predominant ST was ST131 (n = 47, 36.2 %), followed by ST405 (n = 19, 14.6 %), ST38 (n = 15, 11.5 %), ST648 (n = 9, 6.9 %), and ST10 (n = 8, 6.2 %, 8). These 5 majors STs accounted for 75.4 % (n = 98) of all studied isolates. Major STs were distributed in both hospital and community isolates and ST131 was the most common type, not only in the community but also in the hospital. Moreover, ST131 clone was a more frequent cause of ESBL-producing *E. coli* infections/colonizations in the hospital than in the community (*P* = 0.034, OR 2.19, 95 % CI 1.06-4.53). There were no major STs that produced a specific CTX-M type exclusively; *E. coli* isolates of the diverse STs produced CTX-M-15 or CTX-M-14 or both CTX-Ms. However, ST131 isolates were significantly associated with the production of CTX-M-15 (39/47, 82.9 %), either alone (n = 27, 57.4 %; *P* < 0.001) or combined with CTX-M-14 (n = 12, 25.5 %; *P* < 0.001), when compared to non-ST131 isolates. Furthermore, ST131 isolates were more likely to produce OXA-1 concomitantly (29/47, 61.7 %), compared to the non-ST131 isolates (20/83, 24.1 %) (*P* < 0.001, OR 5.08, 95 % CI 2.34-11.00). There was no difference in MDR rates between ST131 (85.1 %, 40/47) and non-ST131 isolates (87.9 %, 73/83). However, ST131 was more likely to be non-susceptible to multiple antimicrobial classes than non-ST131, as 61.7 % of ST131 isolates were non-susceptible to ≥ 3 antimicrobial classes other than penicillin and cephalosporin, versus 36.8 % (7/19) of S405 isolates and 33.3 % (5/15) of ST38 isolates. Particularly, the ST131 clone producing CTX-M-15 (28/39, 71.8 %), either alone or together with CTX-M-14, were more likely to be non-susceptible to ≥ 3 antimicrobial classes, apart from penicillin and cephalosporin, than other genotypes, including ST131 producing CTX-M-14 (1/8, 12.5 %), other STs producing CTX-M-15 (16/29, 55.2 %), either alone or coupled with CTX-M-14, and other STs producing CTX-M-14 alone (22/54, 40.7 %).

**Table 4:** **Multilocus sequence typing of CTX-M-producing* Escherichia coli *isolates**

**ST (allelic profile)**^**a**^	**Total(n = 130)**	**Location of acquisition**		**CTX-M type**		
		**Hospital(n = 56)**	**Community(n = 74)**	**M-14(n = 62)**	**M-15(n = 52)**	**M-14 + M-15 (n = 16)**
131 (53-40-47-13-36-28-29)	47	26	21	8	27	12
405 (35-37-29-25-4-5-73)	19	8	11	13	4	2
38 (4-26-2-25-5-5-19-4)	15	7	8	10	4	1
648 (92-4-87-96-70-58-2)	9	5	4	2	7	
10 (10-11-4-8-8-8-2)	8	1	7	7	1	
69 (21-35-27-6-5-5-4)	4		4	4		
95 (37-38-19-37-17-11-26)	4		4	1	3	
14 (14-14-10-14-17-7-10)	3	2	1	2	1	
354 (85-88-78-29-59-58-62)	3	1	2	3		
410 (6-4-12-1-20-18-7)	3	1	2	2	1	
1177 (4-26-2-211-5-5-19)	3	1	2	1	1	1
1193 (14-14-10-200-17-7-10)	2	1	1	1	1	
2003 (4-26-2-25-4-5-19)	2		1	2		
12 (13-13-9-13-16-10-9)	1		1	1		
224 (6-4-33-16-11-8-6)	1		1	1		
448 (6-6-5-16-11-8-17)	1		1	1		
641 (9-6-33-131-24-8-7)	1		1		1	
2004 (4-26-47-25-36-5-29)	1	1		1		
2656 (35-37-47-25-4-5-73)	1	1		1		
2657 (53-40-2-13-36-28-29)	1		1		1	
2658 (4-26-29-211-5-5-19)	1	1		1		

^a^Alleleic profile, *adk-fumC-gyrB-icd-mdh-purA-recA*.

### Pulsed field gel electrophoresis

PFGE was performed on 130 isolates. Using a > 80 % similarity cut-off point, PFGE analysis showed the genetic heterogeneity in CTX-M-producing *E. coli* isolates, revealing 75 distinctive PFGE types. Only 9 PFGE types were identified to contain 3 isolates or more, which comprised 43.1 % of all studied isolates. PFGE type 10 had the largest number of isolates (n = 18, 13.8 %), followed by PFGE type 63 (n = 9, 6.9 %), type 32 (n = 7, 5.4 %), type 14 (n = 6, 4.6 %), and 3 types (8, 9, and 18) including 3 isolates each. At the 70 % similarity, several PFGE types were grouped into three large clusters; PFGE type 8 to 16 (34 isolates) clustered as Cluster A; PFGE type 63–65 (12 isolates) as Cluster B; PFGE type 32 (7 isolates) as Cluster C. Each cluster was composed of isolates mainly belonging to the specific ST; isolates in Cluster A were identified as ST131, Cluster B as ST405, and Cluster C as ST648. Isolates belonging to each cluster were distributed in both the community and the hospital. PFGE types of isolates belonging to ST38 or ST10 were highly diverse.

**Figure 1:**
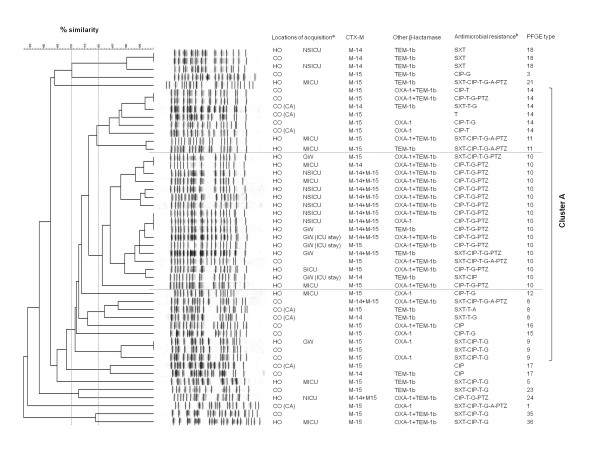
**Dendrogram of extended spectrum β-lactamase (ESBL)-producing *Escherichia coli * isolates belonging to ST131.** Dendrogram based on *Xba*I macrorestriction patterns of EBSL-producing *E. coli* isolates belonging to ST131. The broken vertical lines indicate 80 % and 70 % similarity of PFGE profiles, respectively. ^a^Locations of acquisition: HO, hospital-onset; CO, community-onset; CA, community-associated; MICU, medical intensive care unit; NSICU, neurosurgical intensive care unit; SICU, surgical intensive care unit; GW, general ward. ^b^Antimicrobial resistance denotes antimicrobial agents to which *E. coli* isolates were non-susceptible, apart from penicillin and cephalosporin: SXT, trimethoprim-sulfamethoxazole; CIP, ciprofloxacin; T, tobramycin; G, gentamicin, A, amikacin; PTX, pipercillin-tazobactam.

The ST131 isolates were genetically diverse on PFGE analysis, comprising 18 different PFGE types, and showed an overall similarity of 53 % (Figure 1.). Nonetheless, PFGE analysis within ST131 isolates illustrated that 34 (72.3 %) of 47 ST131 isolates clustered into Cluster A. Among these 34 isolates, 17 ST131 isolates belonged to a single genotype, PFGE type 10, and they were characterized by multidrug resistance (n = 17, 100 %) and producing both CTX-M-14 and -15 in association with OXA-1 or TEM-1b (n = 10, 58.8 %). Furthermore, 94.1 % of these isolates were non-susceptible to ≥ 3 antimicrobial classes other than penicillin and cephalosporin. All of these isolates were recovered from patients with hospital-onset infections (n = 16) or a patient with previous history of admission to this hospital. Thus, these findings suggested that the dissemination of this clone (ST131-PFGE type 10) with multidrug resistant phenotype was responsible for the outbreak at this hospital. Moreover, two PFGE types in Cluster A were identified only in the community, indicating the clonal spread also in the community.

## Discussion

In this study, *E. coli* produced ESBLs with CTX-M type enzymes and all of them were identified as CTX-M-14 or CTX-M-15 or as a combination of both. CTX-M-14 was the predominant enzyme in both the community and the hospital isolates, followed by CTX-M-15. Although there was no significant difference in the prevalence of CTX-M-14 and CTX-M-15 in the community and the hospital isolates, the proportion of CTX-M-14 was higher in community isolates with being more prominent among ESBL-producing *E. coli* infections/colonizations in patients without any healthcare risk factors, i.e. community-associated. Despite the predominance of CTX-M-14 in the community, it was notable that the overall prevalence of CTX-M-15 (38.1 %) increased compared to the earlier Korean studies performed on *E. coli* isolates collected in 2005 (8/47, 17.0 %) [11,12]. Moreover, the more recent studies showed that in Korean hospitals, CTX-M-15-producing *E. coli* isolates outnumbered CTX-M-14 producers [13]. Furthermore, in the community, CTX-M-15 was identified as frequently as CTX-M-14 (39/101, 38.6 % *vs.* 40/101, 39.6 %) in the year of 2010 [15]. This changing epidemiology of CTX-M type ESBLs was also reported in Canada, where CTX-M-14 used to be the most prevalent enzyme in early the 2000s, but was substituted by CTX-M-15 in recent years [31].

The rapid emergence of CTX-M-15-producing *E. coli* worldwide is considered to be attributed to the dissemination of mobile genetic elements, as well as the spread of specific clones, predominantly the international clone ST131 [3,10]. The ST131 clone producing CTX-M-15 is a worldwide pandemic clone, causing predominantly community-onset infections. Its pandemic spread was identified in 2008 by using MLST of CTX-M-15 producing *E. coli* from three continents [32]. In the early studies, the ST131 clone that produced CTX-M-15 had the propensity to cause community-onset infections, especially urinary tract infections [5-7]. However, this clone, in recent times, has also been identified in isolates recovered from the healthcare settings such as the hospital and the nursing homes [8,9].

In this study, ST131 was the most prevalent clone in both the community and the hospital as in the previous reports. Besides ST131, other STs (ST405, ST38, ST648, and ST10) were also identified as a major clone, and all of them were also found to be distributed in both the community and the hospital environments. Interestingly, these 5 major STs, in this study, were also the major clones among CTX-M producing *E. coli* in Canada [8]. ST405, in particular, has been shown to be important in the global spread of CTX-M-15 producing *E. coli*[4]. A previous study in Korea demonstrated that a large proportion of CTX-M-15 producing *E. coli,* collected in 2008, belonged to ST405. This suggested its role for disseminating CTX-M-15 in Korea [13]. In this study, *E. coli* isolates of different STs were found to produce CTX-M-14 or CTX-M-15 or both, and thus, there were no exclusive major clones confined to one specific CTX-M type. This indicates that incorporation of CTX-M-14 or CTX-M-15 might not prefer particular clone, and horizontal transfer of resistance genes among different clones is responsible for dissemination of CTX-M ESBLs in this region. However, ST131 clone was significantly associated with CTX-M-15, either alone or coupled with CTX-M-14, while ST405 and ST38 had tendency to produce CTX-M-14. In addition, we observed the dissemination of several clones producing the same CTX-M type enzyme in the hospital, in the community, and in both environments across the boundary between the hospital and the community. Therefore, the clonal spread of epidemic clones producing CTX-M-15 or CTX-M-14 also played a key role in emergence of ESBL-producing *E. coli* in this region in Korea.

The successful clonal expansion of the ST131 clone producing CTX-M-15 was postulated to be promoted by their distinctive genetic characteristics. ST131 clone appears to harbor a broad range of virulence genes and other resistance genes, such as TEM-1, OXA-1, and a variant aminoglycoside modifying enzyme, *aac(6’)-Ib-cr* on transferable plasmids [3]. This virulence-resistance combination might give them more competitive edge over other *E. coli* isolates [33]. In this study, the ST131 isolates producing CTX-M-15 exceeded other genotypes for the extent of antibiotics resistance. They also frequently co-produced OXA-1 and TEM-1b, and the co-production of OXA-1 was significantly higher in these isolates than others. Of note, we identified a hospital outbreak caused by a group of ST131 isolates which were genetically related (belonging to PFGE type 10), for 10 months during the study period. Among 17 isolates belonging to ST131-PFGE type 10, 10 isolates produced both CTX-M-14 and CTX-M-15 together with OXA-1 and TEM-1b, presenting the similar multidrug resistance profile. We observed the detection of these isolates in the patients who mainly stayed in the intensive care units (ICUs), where the antibiotic pressure is high, before or at the time of culture. This distinctive clone was presumed to evolve carrying multiple β-lactamase genes during the process of adaptation to the hospital environments of high antibiotics pressure, making it possible to successfully disseminate in the ICUs.

Although the coexistence of a CTX-M with other types of ESBL is no longer a rare event, the concomitant presence of 2 CTX-Ms in the same isolate is rarely reported [21,34]. Interestingly, despite the low prevalence, most isolates producing both CTX-M-14 and -15 have been reported in Korea [14,15,35]. It is difficult to point out the possible reason for this phenomenon. Therefore, a continuous study on the molecular characteristics of ESBL-producing *E. coli* is warranted in Korea. In this study, a relatively large number of isolates were found to carry both CTX-M-14 and -15, and this appears to result from the clonal spread of a specific ST131 clone carrying both CTX-Ms at this hospital. However, ST131 was not the only clone that produced both CTX-M-14 and -15 concomitantly. ST405, ST38, and ST1177 also represented *E. coli* isolates producing both CTM-M-14 and -15. Thus, in addition to the clonal spread, the horizontal transfer of plasmids carrying both CTX-M-14 and -15 genes might play a certain role in the hospital dissemination of *E. coli* isolates producing both CTX-Ms. Taken that OXA-1 and TEM-1b were frequently co-produced with both CTX-Ms, it was speculated that OXA-1 and TEM-1b could be incorporated into the same plasmid. Furthermore, multidrug-resistant phenotypes of these isolates raised the possibility of existence of other resistance genes on the same plasmid or in this specific clone, which prompted the further elucidation on molecular characteristics of these strains in the future.

The limitation of this study, in nature, was that it was performed at a single institution in Korea. Thus, the study results may not reflect the epidemiology of different centers and/or different geographic areas. Despite this limitation, this is the first study describing the genetic characteristics of ESBL- producing *E. coli* causing community-onset infection/colonization in comparison with the hospital isolates in Korea to our knowledge. In addition, study results supported the evidence that the rising prevalence of CTX-M-15-producing *E. coli* not only in the community but also in the healthcare settings, and the predominance of ST131 responsible for clonal expansion of ESBL-producing *E. coli* in Korea, which are consistent with the changing epidemiology of ESBL-producing *E. coli* worldwide. Furthermore, this study was conducted at a community hospital, which can reflect the epidemiological changes in the Korean community more than other previous studies conducted a large tertiary referral centers.

## Conclusions

We found that ST131 clone producing CTX-M-15 was the predominant clone among ESBL-producing *E. coli* isolates, not only from the community but also from the healthcare settings in Korea. Besides the ST131 clone, ST405 and ST38 also emerged as important *E. coli* clones spreading CTX-M type ESBLs in Korea and these major clones were identified across the boundary between the hospital and the community. From this study, we found that the rapid emergence of ESBL-producing *E. coli,* in this region, was due to a complicated process boosted by the high transferability of CTX-M type genes. Among several major clones, ST131 showed predominance, which effectively disseminated in the community and the hospital. The ST131 clone was presumed to have a potent ability to acquire various resistance determinants, such as multiple CTX-Ms in this study, which might give it the competitive edge over the other *E. coli* clones in environments of high antibiotics pressure. As the barrier between the hospital and the community has become blurred, in regards to ESBL-producing *E. coli*, as illustrated in this study, the ST131 clone producing multiple CTX-Ms with multidrug resistant phenotypes could have the potential to effectively spread even in the community if they were introduced into the community. Therefore, it is needed to continuously monitor the molecular characteristics of ESBL-producing *E. coli* and to conduct a future investigation for the transmission dynamics on a nationwide scale.

## Competing interests

The authors declare that they have no competing interests.

## Authors' contributions

SHP was involved in all processes related to study design, collecting samples and reviewing medical records, analyses of data, and wrote this paper. JHB and CMP contributed to microbiological and molecular studies. CSM, DGL, SHK, JCK, and JHC contributed to this study by reviewing and making comments on all drafts of this paper. JHY reviewed and revised this paper, and gave final approval to submit for publication. All authors have read and approved the final manuscript.

## Pre-publication history

The pre-publication history for this paper can be accessed here:

http://www.biomedcentral.com/1471-2334/12/149/prepub
